# The Improved Dual-view Field Goniometer System FIGOS

**DOI:** 10.3390/s8085120

**Published:** 2008-08-28

**Authors:** Jürg Schopfer, Stefan Dangel, Mathias Kneubühler, Klaus I. Itten

**Affiliations:** University of Zurich, Remote Sensing Laboratories, Winterthurerstrasse 190, 8057 Zurich, Switzerland E-mails: dangel@geo.uzh.ch, kneub@geo.uzh.ch, klaus.itten@geo.uzh.ch

**Keywords:** Dual-view field goniometer system FIGOS, BRF retrieval, BRDF, atmospheric influence, diffuse illumination, spectrodirectional Remote Sensing

## Abstract

In spectrodirectional Remote Sensing (RS) the Earth's surface reflectance characteristics are studied by means of their angular dimensions. Almost all natural surfaces exhibit an individual anisotropic reflectance behaviour due to the contrast between the optical properties of surface elements and background and the geometric surface properties of the observed scene. The underlying concept, which describes the reflectance characteristic of a specific surface area, is called the bidirectional reflectance distribution function (BRDF). BRDF knowledge is essential for both correction of directional effects in RS data and quantitative retrieval of surface parameters. Ground-based spectrodirectional measurements are usually performed with goniometer systems. An accurate retrieval of the bidirectional reflectance factors (BRF) from field goniometer measurements requires hyperspectral knowledge of the angular distribution of the reflected *and* the incident radiation. However, prior to the study at hand, no operational goniometer system was able to fulfill this requirement. This study presents the first dual-view field goniometer system, which is able to simultaneously collect both the reflected and the incident radiation at high angular and spectral resolution and, thus, providing the necessary spectrodirectional datasets to accurately retrieve the surface specific BRF. Furthermore, the angular distribution of the incoming diffuse radiation is characterized for various atmospheric conditions and the BRF retrieval is performed for an artificial target and compared to laboratory spectrodirectional measurement results obtained with the same goniometer system. Suggestions for further improving goniometer systems are given and the need for intercalibration of various goniometers as well as for standardizing spectrodirectional measurements is expressed.

## 1. Introduction

In the field of optical Remote Sensing ground-based goniometer systems are used to position a spectroradiometer into a specific observation position with respect to the target area. The goal is to directly measure the reflected radiation from the target from various observation directions distributed over the whole hemisphere in order to describe the target specific directional reflectance characteristic. This characteristic occurs due to the contrast between optical properties of surface elements and background as well as due to the uneven distribution of illuminated and shadowed areas. The concept, which describes the reflectance characteristic of a specific target area, is called the bidirectional reflectance distribution function (BRDF) [1]. For practical reasons the bidirectional reflectance factor (BRF) is used and defined as the BRDF of the target ratioed to the BRDF of an ideal Lambertian surface (1/π) [1]. Accurate knowledge of the surface BRF is important for many applications such as BRF correction of remote sensing data and quantitative retrieval of vegetation [2-4], snow [5] or soil [6] parameters. Furthermore, BRF knowledge supports the determination of the surface albedo, which is a crucial parameter in modeling the Earth's radiation budget. The surface albedo is defined as the directional integration of reflectance over all sun-view geometries. Practically, an estimate of the albedo is inferred from the measured nadir reflectance since corresponding satellite sensors often operate at only one or a few view angles [7-9]. Consequently, the surface BRF often is not considered which may lead to large errors in the retrieved albedo [10, 11] and subsequent climate models.

Early goniometer systems were used to measure reflectances of rock samples, soil powders and snow to explain the scattering properties of the surface of the moon [12-14]. The reflectance properties of single plant leaves were first studied using small target goniometers [15-18] and later, larger goniometers have been developed to investigate the reflectance characteristics of soil surfaces and vegetation canopies, e.g. [19-24]. Such ground level spectrodirectional measurements can be performed either in the field [25, 26] or in a laboratory environment [27, 28]. However, there are obvious technical differences between the two concepts and corresponding measurements are not directly comparable [29].

Laboratory measurements provide a better control of the illumination conditions and the presence of diffuse light can be neglected if the experiment is conducted in a darkroom [30]. However, they suffer from illumination imperfections since the artificial light source shows a conical rather than directional geometry leading to an inhomogeneity of the illuminated area. Therefore, the measured reflectance quantity in the laboratory is called biconical reflectance factor (BCRF) corresponding to a conical illumination and observation (FOV) geometry. For the laboratory case, an accurate BRF retrieval (correction for illumination imperfections) is described by Dangel *et al.* [29].

Field goniometry has the advantage that the target is left in its natural environment, including the natural illumination by the sun. The major disadvantage is that atmospheric effects and undesired time variations of the illumination have to be taken into account. Furthermore, the total illumination involves all directions within the hemisphere (solid angle equals 2π) and consists of a diffuse and a direct part. By contrast, observation sensors usually collect the reflected radiation within a certain solid angle and of a small finite area. The atmospheric conditions, the presence of gases, clouds, and aerosols affect the amount and spectral distribution of the incoming direct and diffuse light and cannot assumed to be isotropic and uniform throughout the hemisphere. The measured field reflectance quantity is therefore referred to as hemispherical conical reflectance factor (HCRF) based on Martonchik *et al.* [31] and Schaepman-Strub *et al.* [32]. Consequently, the measured HCRF needs to be corrected for the atmospheric influence in order to obtain the target specific BRF.

The most exact BRF retrieval from field goniometer measurements can be achieved by following the procedures proposed by Martonchik and others [33, 34]. However, this implies accurate knowledge of the angular distribution of the incoming diffuse radiation at the same time as reflected radiation from the target is collected. Most goniometer measurement setups do not account for this. With the Portable Apparatus for Rapid Acquisition of Bidirectional Observations of Land and Atmosphere (PARABOLA III) such data can be collected to a certain degree, but over a limited spectral range only (multispectral) and under the assumption of an extensive homogeneous target area [35]. Another instrument providing such capability consists in the Gonio Radiometer Spectrometer System (GRASS), which is currently being developed at the National Physical Laboratory (NPL), Teddington, UK [36]. It shows a promising and novel dual view design, but has not yet reached an operational status.

Consequently, and prior to the study at hand, there existed no adequate instrument and operational measurement setup, which was capable of observing the reflected and incoming diffuse radiation simultaneously at high angular and spectral resolution. Therefore, no systematic field-laboratory comparison of retrieved BRF of the same target can be performed and it is not known how field measurements can be transferred to laboratory measurements and for which targets a replacement of field by laboratory experiments is indeed feasible.

This study presents the first hyperspectral dual-view field goniometer system (dual-view FIGOS), which is able to simultaneously obtain the reflected and the incoming diffuse radiation at high angular resolution. A characterization of the angular distribution of the incoming diffuse illumination is presented for several atmospheric conditions along with the field BRF retrieval for an artificial target. The dual-view FIGOS showed a stable and reliable performance during several extensive measurement campaigns and strongly supports future surface BRF generation being used for e.g. model validation and inversion purposes as well as for albedo calculations. Additionally, its combined use with multiangular spaceborne or airborne data acquisition provides the possibility of improved directional calibration instead of using nadir-view surface measurements for verification.

## 2. Dual-view field goniometer FIGOS

The presented dual-view field goniometer system is based on the well known FIGOS system (FIeld GOniometer System), which was originally constructed by W. Sandmeier at Lehner & Co. AG, Gränichen, Switzerland, in joint operation with the Remote Sensing Laboratories (RSL) at the University of Zurich, Switzerland [22]. It is a transportable system and has extensively been used in various campaigns for the acquisition of hyperspectral directional reflectance data of vegetation [3, 26, 37, 38], snow [39] and artificial [40] targets. Over the years the capabilities of RSL's goniometer system have been extended in order to support the accurate characterization of the reflectance properties of specific targets in the laboratory as well as in the field. For a description of the laboratory setup please refer to Dangel *et al.* [29].

**Figure 1.**
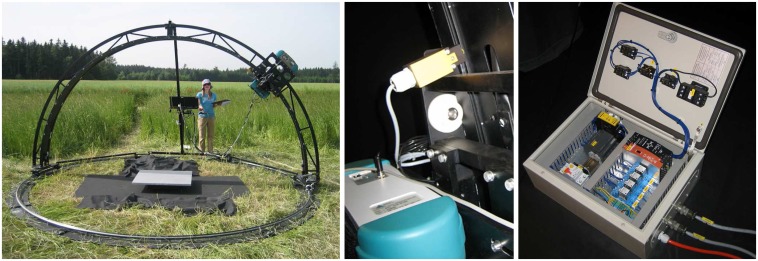
Left: Dual-view goniometer system FIGOS. Middle: Mechanical positioning sensors. Right: Electrical control unit of the step motor.

The goniometer itself consists of three major parts: a zenith arc and an azimuth rail, each of 2 m radius, and a motorized sled, onto which the two sensors are mounted. All parts are made of black-coated aluminum in order to minimize adjacency effects. The zenith arc is tightly fixed to four wagons which allow a manual 360° rotation on the azimuth rail. A braking motor at a velocity of 2.5°/s drives the sled with the two spectroradiometers. Fully adjustable labels on the zenith arc allow for an automated positioning of the spectroradiometers at desired steps. The mechanical positioning sensors as well as the electrical control unit of the motor were renewed in order to resist humidity and guarantee a stable performance. Currently, measurements are taken at azimuth steps of 30° and zenith steps of 15° (-75° to 75°). A full dual-view goniometer dataset is completed in about 25 minutes. Figure 1 shows the dual-view goniometer FIGOS being used for data collection over an artificial target and a close-up of the positioning sensors and the electrical control unit.

### 2.1. Dual-view combination

**Figure 2.**
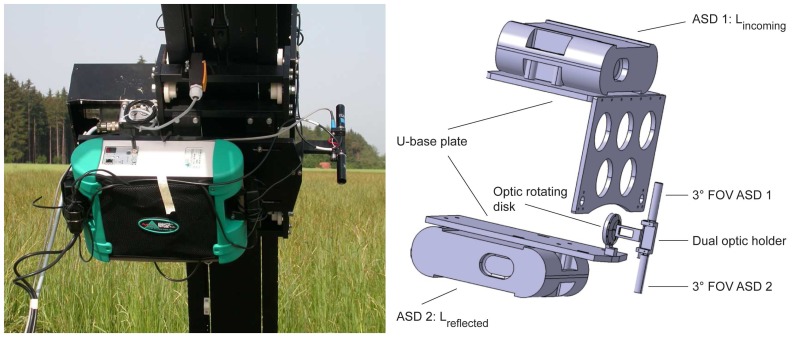
Dual-view combination as mounted onto the zenith arc (left) and corresponding technical sketch (right).

The main extension for the field usage consists of a dual-view combination providing the capability to *simultaneously* collect the reflected and incoming radiances at high spectral *and* high angular resolution. Two wirelessly computer controlled ASD FieldSpec-3 spectroradiometers cover the spectral range from 350 nm to 2,500 nm and sample data at intervals of 1.4 nm (350 – 1,050 nm) and 2 nm (1,000 – 2,500 nm) with a spectral resolution of 3 nm at 700 nm and 10 nm at 1,400/2,100 nm, respectively [41]. Both spectroradiometers are mounted onto the zenith arc of the goniometer and operated with a 3° FOV foreoptic which is connected to the sensor using a 1.4 m fibre optic. The downward looking spectroradiometer observes the target from a constant distance of 2 m for all observation directions. The idea of having both instruments being moved while taking directional measurements evolved from various considerations. The design of a U-base plate (see Figure 2) supports the attachment of both spectroradiometers as closely as possible to the zenith arc. Therefore, and since the zenith arc is eccentrically positioned, no cast shadow is generated on the target area (except for the dual optic holder at the hotspot direction), even though a large volume is moved along the zenith arc. Additionally, fibre optics of standard length can be used and a sufficient signal to noise ratio (SNR) is obtained. In contrast, having only the optics moved (and the spectroradiometers placed outside the goniometer) would create the need of having very long fibre optics (> 4m) and consequently a lower SNR.

By using a dual optic holder both optics are exactly aligned while pointing in opposite directions and the generated shadow at the hotspot direction is minimized to the optic's size, which is about 1cm in diameter. Consequently spectrodirectional measurements close to the hotspot are possible and may provide new insights into the reflectance characteristic of specific targets at this special observation direction. The optic rotating disk allows for easy and quick rotation of the dual optic holder, if necessary, for e.g. additional reference measurements in the beginning of each zenith arc cycle or instrument optimization purposes. Figure 2 shows the U-base plate carrying both spectroradiometers and the dual optic holder.

Since the instantaneous FOV is 3° and always pointing to the centre of the hemisphere (downward looking optic), the corresponding ground instantaneous field of view (GIFOV) is circular with 10.5 cm (diameter) in nadir direction. However, for large off-nadir observation angles the sensor's footprint becomes elliptical with a maximum longitudinal extent of 41cm for an observation angle of 75°. It is therefore essential to consider the correct target reference height, especially when measuring a target with limited size e.g. under laboratory conditions.

**Figure 3.**
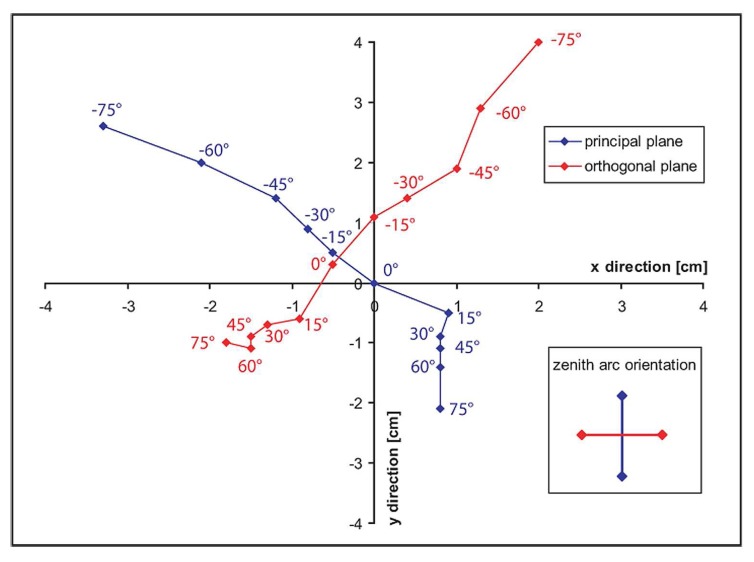
Pointing accuracy over the zenith arc. The convention −x/y and +x/y is used for the backward scattering and the forward scattering direction, respectively. The coordinate system is aligned to the centre of the azimuth arc.

In order to monitor the pointing accuracy of the downward looking optic, a small laser is integrated into the dual optic holder. The geometric precision of the zenith arc is then referenced while moving the sled over the zenith arc in the principal and in the orthogonal plane. Maximum deviation of the laser spot, representing the centre of the sensor GIFOV, is recorded at a view angle of -75° and consists of about 4cm as shown in Figure 3. A possible cause for this deviation might be a slight deformation of the respective part of the zenith arc due to extensive usage (assembly/disassembly) over time. However, this is not a limiting factor for field goniometer measurements since the target under observation is usually of satisfying spatial extent and assumed to be homogeneous.

### 2.2. Measurement principle

Spectrodirectional measurements with the dual-view FIGOS usually start in the principal plane at a forward scattering direction of 75°. Following a predefined sequence the whole hemisphere is scanned at zenith steps of 15° and azimuth steps of 30°. Spectralon references are collected in the beginning and in the end of each goniometer dataset as well as at every nadir bypass with the downward looking sensor. This provides the potential of calculating reflectances, if wished at a later time, and of monitoring atmospheric changes or instrument drifts. In total 140 measurements are taken for one dual-view goniometer dataset (8 reference measurements plus 66 directional measurements of the reflected and incoming radiances, respectively).

Even though shadowing is minimized it might occur anyway when the sun zenith angle equals one of the (downward looking) sensor view angle steps (e.g. at 15°, 30°, 45°, 60° or 75°). If this is the case, the corresponding measurements are omitted, interpolated, or modelled by fitting to a BRF model.

Simultaneous sunphotometer measurements are necessary for two reasons: 1) monitoring the state of the atmosphere during the whole measurement time and 2) the direct sun irradiance is required as an input parameter to the field BRF retrieval algorithm. Test measurements to collect the direct sun irradiance using the upward looking sensor revealed saturation problems of the sensor. Although this problem might be solved by reducing the integration time of the upward looking spectroradiometer, the dual-view FIGOS is currently not yet able to directly measure this quantity. This is mainly due to the fact that using a 3° FOV accurate pointing at the sun disk is challenging and time consuming. The time for measuring one goniometer dataset is a critical factor and desired to be as short as possible.

Within the current measurement setup an MFR-7 shadowband sunphotometer (Yankee Environmental Systems, Inc) is used, which directly records the total and diffuse irradiance in 7 bands (broadband, 415, 500, 615, 673, 870 and 940 nm). The direct sun irradiance is then calculated as a difference of the two, taking the respective sun zenith angle into account.

## 3. Data processing

**Figure 4.**
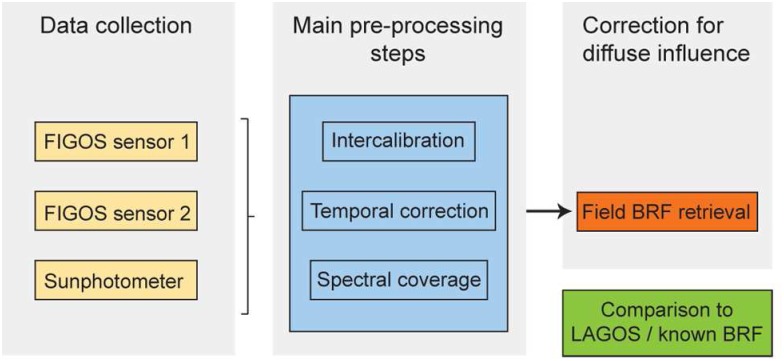
Dataflow scheme for processing dual-view FIGOS datasets.

A number of pre-processing steps have to be performed prior to conducting the actual BRF retrieval. These steps are directly related to the current goniometer systems' characteristics. They include the calculation of intercalibration coefficients, a temporal correction and an assessment of the different spectral coverage. A detailed description of the main pre-processing steps is given below. Figure 4 gives an overview of the dataflow for processing dual-view FIGOS datasets.

### 3.1. Intercalibration

The need for intercalibration coefficients evolves from the fact that currently three spectroradiometers are used to obtain the necessary input data to the BRF retrieval algorithm. The angularly resolved reflected and incoming diffuse radiation is obtained with two separate instruments, although of the same type (ASD FieldSpec 3). The direct irradiance is obtained from sunphotometer measurements (MFR-7 shadowband sunphotometer).

#### A. ASD FieldSpec 3 intercalibration

**Figure 5.**
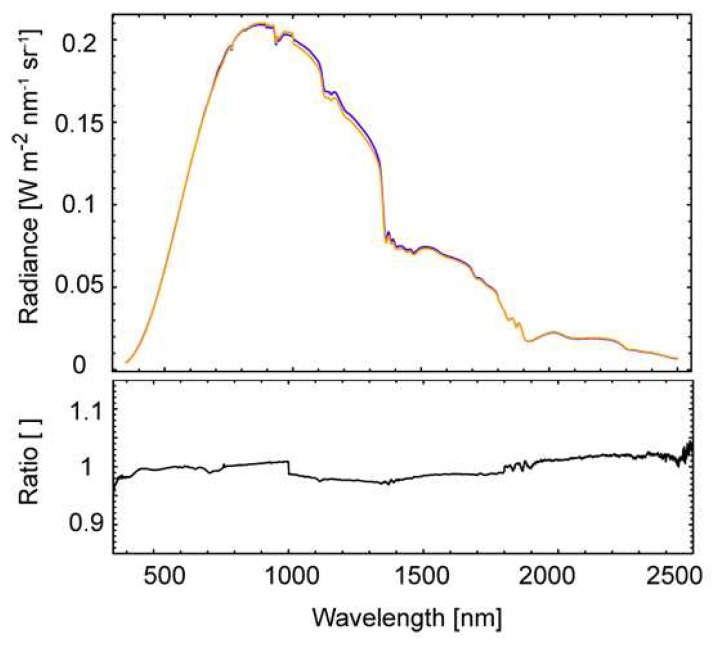
Comparison of absolute radiance values and intercalibration coefficients for the two ASD FieldSpec3 sensors.

The two spectroradiometers, which are used to simultaneously collect the reflected and incoming diffuse radiation, are usually operated in radiance mode. For further processing, the intercalibration coefficients have to be known for the two instruments. The last intercalibration experiment with the two FIGOS spectroradiometers has been performed in July 2006 at the intercalibration facility of the German Aerospace Centre (DLR) offering an integrating sphere with the corresponding infrastructure for stable conditions [42]. Figure 5 shows a comparison of the absolute radiance values as measured with the two ASD FieldSpec3 as well as the current intercalibration coefficient. The agreement for the VNIR detector is within 1%, whereas for the SWIR1 and SWIR2 detectors it consists of about 2%. Extreme values at both ends of the spectral range reach up to 4%.

#### B. ASD FieldSpec 3 – sunphotometer intercalibration

These intercalibration coefficients are obtained by comparing the directly measured hemispherical irradiance values from sunphotometer measurements E_mfr_(θ_i_) and the hemispherical irradiance values E_asd_(θ_i_) retrieved from Spectralon nadir measurements performed with the ASD FieldSpec 3 for certain solar zenith angles θ_i_. For deriving E_asd_(θ_i_), the Spectralon panel is either assumed to be Lambertian or, more accurately, a BRF correction factor has to be taken into account. Assuming a Lambertian behaviour of the Spectralon panel the hemispherical irradiance E_asd_(θ_i_) can be derived from a single measurement of the reflected radiation L_r_ taking the albedo ρ_ls_ of the calibration protocol into account and is written as

(1) $${\text{E}}_{\text{asd}}(\theta_{\text{i}})=\frac{{\text{L}}_{\text{r}}(\theta_{\text{r}},\varphi_{\text{r}})*\pi }{\rho_{\text{ls}}}.$$

Provided the knowledge of E_mfr_(θ_i_) and E_asd_(θ_i_) the respective intercalibration coefficient can then be written as

(2) $${\text{c}}_{\text{mfr}}(\theta_{\text{i}})=\frac{{\text{E}}_{\text{mfr}}(\theta_{\text{i}})}{{\text{E}}_{\text{asd}}(\theta_{\text{i}})}.$$

**Figure 6.**
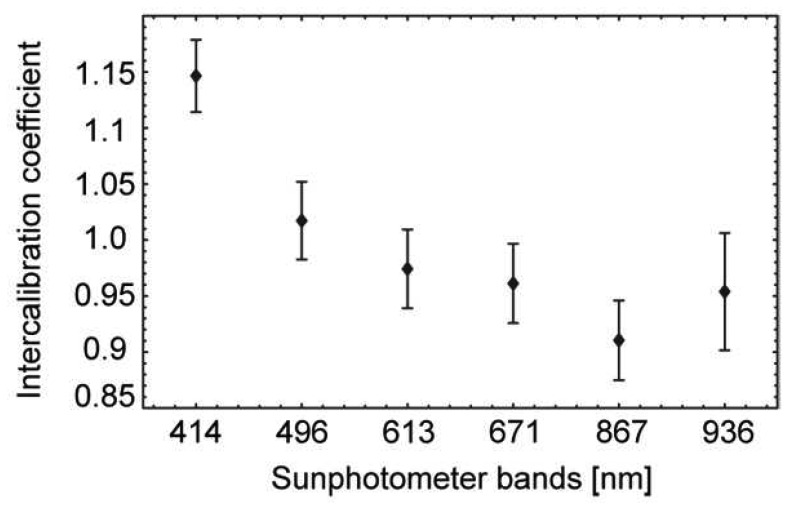
ASD FieldSpec 3 – sunphotometer intercalibration coefficients and respective standard deviation.

The ASD FieldSpec 3 – sunphotometer intercalibration coefficients as shown in Figure 6 for the respective sunphotometer bands represent the averaged values for a solar zenith angle range from 24.7° to 52.9° along with the respective standard deviations.

### 3.2. Assessment of spectral coverage

**Figure 7.**
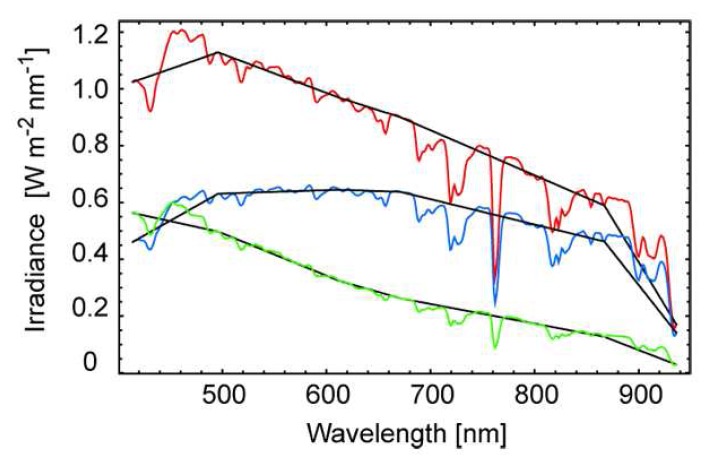
Total (red) and direct (blue) and diffuse (green) irradiance at high spectral resolution versus interpolated sunphotometer measurements (black lines).

Both the reflected and the incoming diffuse radiation are measured with an ASD FieldSpec 3 providing continuous spectral information from 400nm to 2500nm. However, the direct irradiance from the sun is obtained from sunphotometer measurements and is available in six spectral bands from 414nm to 936nm only. Principally, this limits accurate retrieval results to the sunphotometer spectral bands. One might try to obtain a continuous spectral coverage by linear interpolation between the sunphotometer bands, but due to the highly variable atmospheric absorption features this is only a coarse approximation as Figure 7 shows.

A more accurate assessment of the atmospheric absorption features is obtained by weighing the interpolated values for each spectral section between the sunphotometer bands. The respective weight factors are calculated by rationing the interpolated values to E_asd_ (continuous spectral coverage) for the respective solar zenith angles θ_i_. However, in doing so the total irradiance is estimated and not the direct irradiance of the sun. Thus, the ratio of direct to total irradiance from respective sunphotometer measurements needs to be applied to the estimated irradiance values prior to calculating the weight factors. This method is applied to each set of direct irradiance measurements, which are used for the retrieval algorithm (66 measurements per goniometer dataset).

### 3.3. Temporal correction

One goniometer dataset typically consists of 66 measurements for the upward looking as well as for the downward looking sensor. Since the sensors have to be moved between each measurement, these 66 measurements cannot be performed at the same time; the total time period needed consists of about 20 to 25 minutes. Within that time span the illumination conditions do change. This is due to the movement of the sun and due to the changing atmospheric properties (e.g. clouds), which affect the amount and the distribution of the incoming diffuse light. It is tried to account for these effects by weighing the measured incoming diffuse radiance ${\text{L}}_{\text{diff}}^{\text{inc}}$. The weight factors f_diff_ is obtained using the continuous diffuse irradiance readings of the sunphotometer, respectively. Thereby, it is assumed that changes of the diffuse irradiance E_diff,mfr_ within the time period T(t_1_, t_2_, …, t_66_) of a goniometer dataset affect the 66 single incoming diffuse radiation measurements ${\text{L}}_{\text{diff}}^{\text{inc}}$ to a similar degree. The weight factor f_diff_(t_x_) can then be obtained from the ratio E_diff,mfr_(t_x_) / E_diff,mfr_(t_1_) and the incoming diffuse radiation is written as

(3) $${\text{L}}_{\text{diff}}^{\text{inc}}({\text{t}}_{\text{x}})={\text{L}}_{\text{diff}}^{\text{inc}}({\text{t}}_{\text{x}})*{\text{f}}_{\text{diff}}({\text{t}}_{\text{x}}).$$

### 3.4. Field BRF retrieval

Typically, field measurements are affected by atmospheric conditions and underlie a direct and a diffuse illumination component. The distribution of the latter is not necessarily isotropic. Influencing factors are related to the cloud cover, aerosol content and the surrounding area (i.e. forest, hillsides, buildings etc.) which all lead to multiple scattering and a varying amount of incoming diffuse light for each incident direction. However, the observed (reflected) radiance L_r_ at the sensor is the result of the total incoming radiance L_inc_ (both the direct and the diffuse component) interacting with the target specific BRF. In other words, the BRF “tells” the incoming single radiation beams how, meaning how much and in which directions, they are reflected. Physically this is expressed in (4) as follows:

(4) $${\text{L}}_{\text{r}}(-\mu ,\mu_{0},\varphi -\varphi_{0})=\pi^{-1}\int_{0}^{1}\int_{0}^{2\pi }\text{R}(-\mu ,\mu \prime ,\varphi -\varphi \prime )*{\text{L}}^{\text{inc}}(\mu \prime ,\mu_{0},\varphi -\varphi_{0})\mu \prime \text{d}\mu \prime \text{d}\varphi \prime$$

where

- μ, μ0 = cosines of the view and solar zenith angles,
- φ-φ0 = is the view azimuth angle with respect to the solar principal plane and
- R = the BRF of the target.

The notation −μ and μ is used here for upwelling and downwelling radiation, respectively [34]. In order to accurately retrieve the BRF the reflected radiance as well as the single contributors to the incoming radiance field (direct and diffuse radiances) have to be known, preferably with high angular resolution. This can be achieved either by measurements or by modeling. The BRF retrieval for field measurements is performed by following the procedure proposed by Martonchik *et al.* [34]. It is based on the idea of splitting up the radiation into a direct and diffuse part E_dir_ and L_diff_, respectively, and considering their respective reflection processes (the interaction with the surface) separately. The reflected radiance L_r_ is then calculated as

(5) $${\text{L}}_{\text{r}}(-\mu ,\mu_{0},\varphi -\varphi_{0})=\pi^{-1}\text{R}(-\mu ,\mu_{0},\varphi -\varphi_{0})*{\text{E}}_{\text{dir}}(\mu_{0})+{\text{L}}_{\text{diff}}(-\mu ,\mu_{0},\varphi -\varphi_{0})$$

E_dir_ is obtained from sunphotometer measurements and the dual-view FIGOS directly provides spectrodirectional measurements of L_r_. The upward diffuse radiance L_diff_ is also dependant on the surface BRDF (π^-1^R) and is calculated using (6) where the incident diffuse radiance ${\text{L}}_{\text{diff}}^{\text{inc}}$ is directly obtained from dual-view FIGOS measurements.

(6) $${\text{L}}_{\text{diff}}(-\mu ,\mu_{0},\varphi -\varphi_{0})=\pi^{-1}\int_{0}^{1}\int_{0}^{2\pi }\text{R}(-\mu ,\mu \prime ,\varphi -\varphi \prime )*{\text{L}}_{\text{diff}}^{\text{inc}}(\mu \prime ,\mu_{0},\varphi -\varphi_{0})\mu \prime \text{d}\mu \prime \text{d}\varphi \prime$$

The bidirectional reflectance factor R can then be iteratively solved using (5) and the (n-1)^th^ iteration of (6), and is formulated as

(7) $${\text{R}}^{\left(\text{n}\right)}(-\mu ,\mu_{0},\varphi -\varphi_{0})=\frac{{\text{L}}_{\text{r}}(-\mu ,\mu_{0},\varphi -\varphi_{0})-{\text{L}}_{\text{diff}}^{\left(\text{n}-1\right)}(-\mu ,\mu_{0},\varphi -\varphi_{0})}{\pi^{-1}{\text{E}}_{\text{dir}}(\mu_{0})}$$

As an initial estimate of the BRF, R^(0)^ is used where ${\text{L}}_{\text{diff}}^{\text{inc}}$ is neglected and atmosphere-surface reflections are ignored (R^(0)^ = L_r_ / (π^−1^ *E_dir_)). For each iteration, the reflected radiance L_r_ is calculated using the current iteration estimate of R. The iteration is ended when the difference between the calculated and measured reflected radiances, ${\text{L}}_{\text{r}}^{\text{calculated}}$ and ${\text{L}}_{\text{r}}^{\text{measured}}$, respectively, becomes smaller than a previously defined threshold.

## 4. Test study

In order to test the field BRF retrieval based on dual-view FIGOS datasets and quantify the diffuse influence, spectrodirectional field and laboratory measurements have been performed using an artificial target for both cases. The reasons are to minimize differences other than such related to the illumination conditions and to maximize the reflectance anisotropy by choosing an appropriate target.

### 4.1 Target

**Figure 8.**
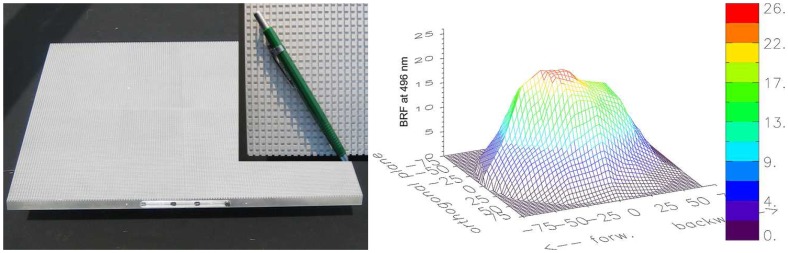
Left: Artificial target, which is being for the BRF retrieval and field – laboratory comparison. Right: Reflectance anisotropy at a wavelength of 496nm as measured in the laboratory (30° illumination direction from the right side).

The artificial target has first been described by Govaerts *et al.* [43] who evaluated a 3D radiative transfer (RT) model against goniometer measurements. The same artificial target has also been tested for its usefulness with FIGOS/LAGOS measurements in earlier studies [40]. The target itself is made of sanded duralumin and consists of a regular matrix of cubes with known geometrical characteristics. It is well qualified for BRF investigations, since it exhibits a high angular anisotropy and is inert over time. However, for FIGOS/LAGOS measurements it was found to be too small since the sensor GIFOV for large observation angles outreached the spatial extent of the target area. Consequently a larger artificial target with similar characteristics has been constructed with the help of the Physics Workshop of the University of Zurich, Switzerland. Its suitability has subsequently been tested in various extensive field and laboratory measurement campaigns [44]. Figure 8 shows the artificial target and its reflectance anisotropy. The size of the cuboids is 3.3 × 3.3 × 3mm with a regular spacing of 2mm between the single cubes.

### 4.2 Dataset

The final data being used for the field BRF retrieval consists of 6 dual-view goniometer datasets (FA1, FA2, FA8, FA9, FA10 and FA11) which were obtained at solar zenith angles θ_i_ ranging from from 24.7° to 52.9°. The data collection took place at three different days (DOY 171, 172 and 175) in June 2006 close to the airport of Oberpfaffenhofen, Germany. The measurement location consisted of a wide, flat area and was carefully selected in order to minimize potential adjacency effects. For comparison purposes corresponding spectrodirectional measurements (same illumination angles) were also performed in the laboratory with the laboratory goniometer system LAGOS.

**Figure 9.**
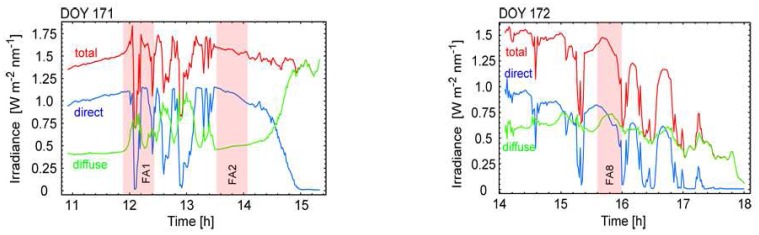

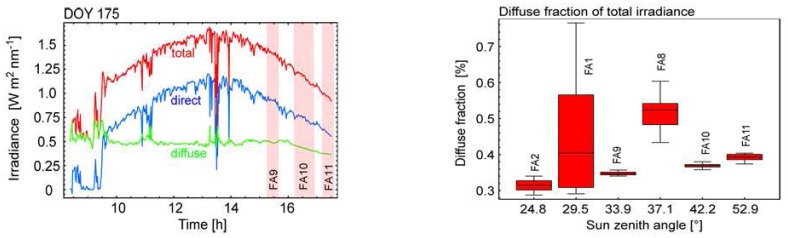
Top and bottom left: Total, direct and diffuse irradiance during the DOYs and the respective goniometer measurement periods. Bottom right: Box plot of the diffuse fraction of the total irradiance for investigated FA datasets. All data are shown for a wavelength of 496nm.

Permanent atmospheric monitoring was assured by using an MFR-7 shadowband sunphotometer. Figure 9 depicts the total, direct and diffuse irradiance components for the respective measurement days as well as a box plot of the diffuse fraction of the total irradiance (E_diff_ / E_tot_) for the 6 goniometer datasets. The size of the boxes represents the interquartile variability (25% - 75% of the values) of the diffuse irradiance during the FIGOS measurement period. The horizontal black lines indicate the median values and the whiskers show the total extent of the dataset. The diffuse fraction significantly varies within the individual datasets. High diffuse variability as seen in datasets FA1 and FA8 is predominantly attributed to passing clouds, whereas the diffuse variability in other datasets is related to the respective sun zenith angle.

### 4.3 Results

#### 4.3.1. Angular distribution of the incoming diffuse radiation

The angular diffuse fractions ${\text{L}}_{\text{diff}}^{\text{inc}}/{\text{E}}_{\text{tot}}$ of the total irradiance were calculated for each measured observation direction. For a clear sky situation the angular diffuse fractions are mainly determined by the solar zenith angle. A major amount of diffuse light is observed close to the sun view direction and minimum values are observed for opposite viewing directions (with the sun in the back). Typically, the angular diffuse fractions also tend to increase for large observation angles since the respective incident light paths are longer and more multiple scattering takes place. For a clear sky situation the angular diffuse fractions reach 15% to 40% depending on the solar zenith angle. A cloudy day situation looks even more complicated. Although a maximum value of the angular diffuse fraction is still observed close to the sun view direction, the distribution of the diffuse light is very much dominated by atmospheric disturbances such as moving clouds. Consequently, the angular diffuse fractions can substantially vary over time and for small changes of the observation direction, and the total angular variability can reach up to about 70%.

**Figure 10.**
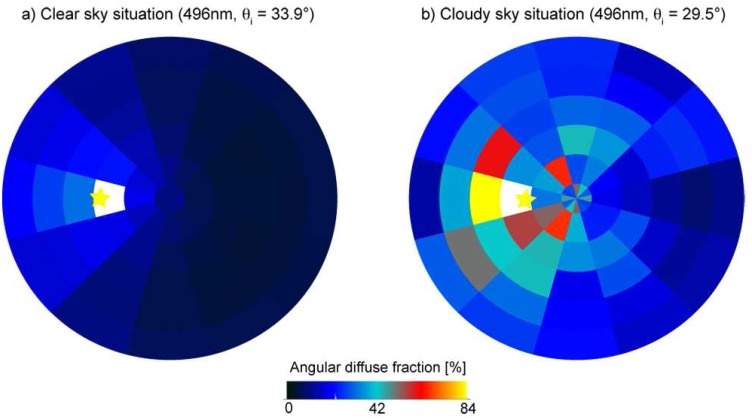
Angular diffuse fraction for different atmospheric situations (clear, cloudy) from dual-view FIGOS measurements (datasets FA9 and FA1). The position of the sun is marked by the sun symbol.

With regard to the BRF retrieval and associated atmospheric correction this highlights the importance of assessing the incoming diffuse radiance at angular resolution even for clear day situations. Figure 10 represents the angular diffuse fractions of the total irradiance for two different illumination atmospheric conditions but a similar solar zenith angle.

#### 4.3.2. Retrieval results

**Figure 11.**
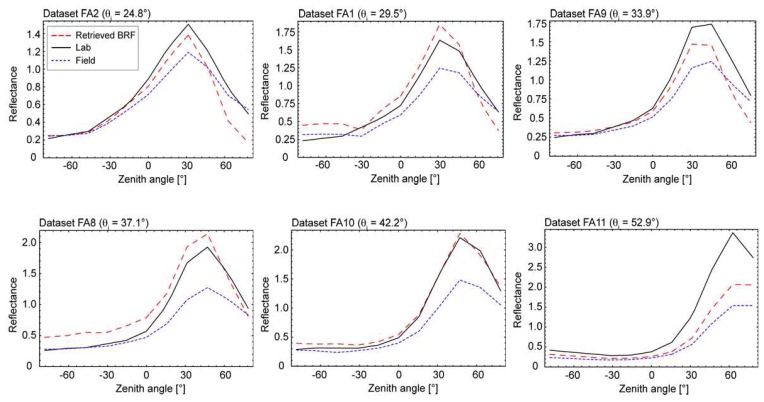
Comparison of BRF retrieval results and spectrodirectional field and laboratory measurements in the principal plane (-75° backward scattering to 75° forward scattering). The illumination is from the left.

The regular geometrical structure of the artificial target leads to a high angular reflectance anisotropy, which strongly correlates with the distribution of the illuminated and shadowed areas for the respective illumination and observation directions. Additionally, due to the optical properties of the sanded duralumin, the artificial target exhibits a strong specular reflectance characteristic. The observed reflectance peak is directly related to the zenith angle of the direct irradiance and is, consequently, moving towards larger observation angles for larger illumination zenith angles. For this particular target largest directional effects are expected in the principal plane. Figure 11 shows the corresponding principal plane reflectance values for observation angles ranging from -75° (backward scattering) to 75° (forward scattering) for the field case, the laboratory case and the BRF retrieval case.

The maximum extent of the specular reflectance peak is obtained at the largest illumination zenith angle (52.9°) and consists of over 300% reflectance for the laboratory case of dataset FA11. It can be seen for all datasets that the directional reflectance characteristic for the laboratory case is more distinct than for the field case. This is due to the fact that the diffuse irradiance incident on the target is illuminating the shadowed areas and mitigating dominant reflectance structures. For vegetation targets Lyapustin *et al.* [33] found that the backward scattering is rather dominated by the direct irradiance whereas changes in the forward scattering are related to the diffuse irradiance. This leads to a lower backward scattering and a greater forward scattering of the field reflectance compared to the BRF. For the artificial target a similar behaviour can partly be identified, which, however, might be superimposed by the strong specular reflectance, which depends on the direct irradiance component. With regard to the retrieved BRF, it can be observed that in general a reasonable approximation to the laboratory reflectance is achieved and for most datasets the specular peak is reproduced well. Best results were obtained for dataset FA10. The largest overall deviations occur for the two datasets, which were obtained at highly variable atmospheric conditions (FA1 and FA8). Remaining differences might be related to the needed time period and to the sampling (oversampling, undersampling) of the incoming diffuse radiances, especially for large observation angles. In laboratory measurements, the specular reflectance might additionally be attenuated by the inhomogeneity of the illuminated area within the GIFOV for corresponding observation zenith angles.

## 5. Conclusions

The dual-view field goniometer system FIGOS is currently the only instrument which is capable of measuring the reflected and the incoming diffuse radiation at the same high angular and at high spectral resolution from 400 nm to 2,500 nm. It showed a stable and reliable performance in extensive field campaigns. In its present configuration, in conjunction with a sunphotometer, it proved its ability to provide the necessary dataset for a field BRF retrieval of selected targets. Therefore, and due to its well-known characteristics the dual-view FIGOS has the potential to being used as a reference instrument for various spectrodirectional experiments in future field campaigns.

Although measurements in both directions are done simultaneously, the critical time to measure a complete goniometer dataset is not increased by having two instruments since both measurements are triggered simultaneously. Simultaneous sunphotometer measurements are needed since the direct solar irradiance (a necessary input to the retrieval algorithm) can currently not be obtained from the upward looking FIGOS sensor. Consequently, a complete retrieval dataset consists of measurements from three different instruments (two of them of the same type). This requires the use of instrument intercalibration coefficients. Since the currently used sunphotometer operates using a limited number of bands only, further pre-processing is necessary in order to obtain spectrally continuous information of the direct irradiance. This is achieved by deriving the atmospheric absorption features between the sunphotometer bands from hyperspectral Spectralon reference measurements. However, hyperspectral measurements of the direct irradiance would substantially ease data pre-processing and provide a larger spectral coverage of the retrieved BRF. A possible solution might be to attach a spectroradiometer to a sun-tracking device in order to continuously collect the solar irradiance. Alternatively, direct irradiance values might also be obtained from simulations using MODTRAN-4 [45].

With the addition of an artificial illumination source (currently a 1,000 W quartz tungsten halogen lamp) the same goniometer system can also be used in a laboratory configuration as LAGOS (without dual-view option). Errors due to inherent system inaccuracies persist but are the same for both goniometer configurations. The usage of an inert, artificial target (for both the field and laboratory experiment) provides the advantage of reducing target related measurement errors and its high angular anisotropy supports FIGOS – LAGOS comparison measurements for e.g. a BRF retrieval. The laboratory setup further provides the possibility of direct comparisons to other goniometer systems currently in use [21, 24, 36, 46, 47].

The time period needed to obtain a complete dual-view goniometer dataset is a critical factor since illumination conditions can rapidly change either because of atmospheric changes (e.g. over passing clouds) or changes in the solar zenith angle (for large solar zenith angles the change ratio is typically larger). Short-term atmospheric variability can affect single diffuse radiance measurements differently, leading to an over- or underestimation of the incoming diffuse irradiance. Using time resolved sunphotometer measurements substantially improved our ability to correcting for illumination changes during the measurement period. Therefore, we propose to rely on the combination of dual-view goniometer measurements and continuous total and diffuse irradiance data acquisition. If, in an ideal case, all directional measurements of the two angular datasets (lower and upper hemisphere) were collected at the same time, this correction would not be necessary. Another way to reduce the influence of atmospheric changes during the time period needed would consists of shortening the time period by changing the measurement sequence or measuring only half of the upper and lower hemisphere assuming a symmetric distribution of the target reflectance and the incident diffuse irradiance.

## 6. Outlook

Although the dual-view FIGOS is well suited for spectrodirectional field data collection and provides a reliable performance, there are also limitations, which need to be assessed. The measurement setup can be further improved with respect to data collection accuracy and ease of use. One possibility consists of assessing the main limitations by reducing the measurement time period, accounting for the changing GIFOV and FOV non-uniformity of the spectroradiometer and collecting the direct irradiance over a continuous spectral range (400 nm – 2,500 nm). However, other drawbacks (e.g. the weight, slight deviations of the zenith arc, pointing accuracy, assembly/disassembly time, etc.) are not accounted for. Another possibility for future development of goniometer systems could consist of a robotic positioning system as carrier for the dual-view sensors (fibre optics). Robotic systems are widely used in industry (e.g. car industry, industrial automated painting etc.) and provide characteristics, which are of great usefulness for goniometers. The spatial positioning of the robotic arm is fully automatic, programmable, very fast and highly reproducible. Furthermore, such systems are easy to handle in the field or in the laboratory and their free programmability allows for highly flexible, target specific angular sampling.

For validation and calibration purposes of air- and spaceborne directional reflectance measurements, as well as for algorithm development and independent RT model validation there is an evolving need for ground-based directional measurements of various surface types. Such ground-based directional data acquisitions must be performed in a standardized and comprehensible way and results as well as metadata need to be well documented and stored in corresponding functional database facilities, e.g. SPECCHIO [48]. Current ground-based spectrodirectional measurement procedures are not standardized and it is unknown how results obtained from various goniometer systems differ. Therefore, it is a further need to perform intercalibration studies with various state of the art goniometers following the example of the modeling community (cf. RAMI [49]). Such studies need to be performed under controlled laboratory conditions using an artificial target. Additionally, it has to be agreed on a common spectrodirectional data format and quality requirements for spectrodirectional measurements have to be defined. Once this is achieved, spectrodirectional measurement results from various campaigns could easily be transferred to such a data standard in order to ensure data comparability between spectrodirectional research groups.
